# *Anisomeles indica* Extracts and Their Constituents Suppress the Protein Expression of ACE2 and TMPRSS2 In Vivo and In Vitro

**DOI:** 10.3390/ijms242015062

**Published:** 2023-10-11

**Authors:** Yu-Ru Chen, Wen-Ping Jiang, Jeng-Shyan Deng, Ya-Ni Chou, Yeh-Bin Wu, Hui-Ju Liang, Jaung-Geng Lin, Guan-Jhong Huang

**Affiliations:** 1 Department of Chinese Pharmaceutical Sciences and Chinese Medicine Resources, College of Chinese Medicine, China Medical University, Taichung 404, Taiwan; yuru880801@gmail.com (Y.-R.C.); yanichoucmu@gmail.com (Y.-N.C.); jglin@mail.cmu.edu.tw (J.-G.L.); 2 Department of Pharmacy, Chia Nan University of Pharmacy and Science, Tainan 717, Taiwan; wpjiang@gm.cnu.edu.tw; 3 Department of Food Nutrition and Healthy Biotechnology, Asia University, Taichung 413, Taiwan; dengjs@asia.edu.tw; 4 Arjil Pharmaceuticals LLC, Hsinchu 300, Taiwan; ybw333@arjilbio.com (Y.-B.W.); kathy@arjilbio.com (H.-J.L.)

**Keywords:** SARS-CoV-2, *Anisomeles indica*, ovatodiolide, anisomlic acid, apigenin, ACE2, TMPRSS2

## Abstract

Coronavirus disease 2019 (COVID-19), stemming from severe acute respiratory syndrome coronavirus 2 (SARS-CoV-2), has had a profound global impact. This highly contagious pneumonia remains a significant ongoing threat. Uncertainties persist about the virus’s effects on human health, underscoring the need for treatments and prevention. Current research highlights angiotensin-converting enzyme 2 (ACE2) and transmembrane protease serine 2 (TMPRSS2) as key targets against SARS-CoV-2. The virus relies on ACE2 to enter cells and TMPRSS2 to activate its spike protein. Inhibiting ACE2 and TMPRSS2 expression can help prevent and treat SARS-CoV-2 infections. *Anisomeles indica* (L.) Kuntze, a medicinal plant in traditional Chinese medicine, shows various promising pharmacological properties. In this study, ethanolic extracts of *A. indica* were examined both in vivo (250 and 500 μM) and in vitro (500 μM). Through Western blotting analysis, a significant reduction in the expression levels of ACE2 and TMPRSS2 proteins was observed in HepG2 (human hepatocellular carcinoma) cells and HEK 293T (human embryonic kidney) cell lines without inducing cellular damage. The principal constituents of *A. indica*, namely, ovatodiolide (5 and 10 μM), anisomlic acid (5 and 10 μM), and apigenin (12.5 and 25 μM), were also found to produce the same effect. Furthermore, immunohistochemical analysis of mouse liver, kidney, and lung tissues demonstrated a decrease in ACE2 and TMPRSS2 protein expression levels. Consequently, this article suggests that *A. indica* and its constituents have the potential to reduce ACE2 and TMPRSS2 protein expression levels, thus aiding in the prevention of SARS-CoV-2 infections.

## 1. Introduction

Designated as coronavirus disease 2019 (COVID-19), this infectious disease emerged in 2019 and is attributed to the severe acute respiratory syndrome coronavirus 2 (SARS-CoV-2). As 2019 was coming to an end, the virus was first detected in Wuhan city and quickly disseminated to various parts of the world [1,2]. Within the coronavirus family, SARS-CoV-2 is characterized by its single-stranded RNA structure. This type of virus typically infects both humans and other animals [3]. While some coronaviruses may only cause mild cold-like symptoms, others, such as SARS (severe acute respiratory syndrome) and MERS (Middle East respiratory syndrome), can lead to more severe diseases. SARS-CoV-2 is characterized by its high contagiousness and is mainly disseminated through respiratory droplets [4]. In situations where an individual who has contracted it coughs, sneezes, or talks, they release respiratory droplets containing a high viral load into the air, which can be inhaled by others, leading to infection [5]. In the human body, SARS-CoV-2 initially infects ciliated cells in the trachea, then proceeds to infect the lower respiratory tract along the trachea and bronchi, ultimately reaching the alveoli. This leads to inflammation and compromised gas exchange [6]. Those afflicted with SARS-CoV-2 can manifest a spectrum of symptoms, varying in intensity from mild to severe. These symptoms encompass fever, cough, difficulty breathing, fatigue, muscle discomfort, and a diminished sense of smell or taste [7,8]. However, severe symptoms typically manifest about a week following the onset of symptoms, with the most common being difficulty in breathing, attributed to hypoxia, eventually progressing into progressive respiratory failure [6].

Research findings suggest that the potency of angiotensin-converting enzyme 2 (ACE2) and transmembrane protease serine 2 (TMPRSS2) could have a pivotal impact on the infectiousness of SARS-CoV-2 [7]. ACE2 is an enzyme widely present in the human body, with its primary function being to regulate the balance of blood pressure and participate in the regulation of the cardiovascular system [9]. Additionally, research has revealed that ACE2 is a crucial receptor for SARS-CoV-2 to enter human cells. The spike protein of SARS-CoV-2 can bind to ACE2 and initiate the mechanism of membrane fusion, allowing the virus to invade cells [10]. On the other hand, TMPRSS2 is primarily responsible for specific protein cleavage and activation on the cell membrane’s surface. Hence, through modification by TMPRSS2, SARS-CoV-2 can penetrate into cells more effectively [11]. Upon entering the cell, SARS-CoV-2 triggers an intense inherent immune reaction, stimulating the secretion of inflammatory cytokines, cell injury, and a procoagulant condition [12]. Previous research has detected ACE2 expression in organs such as the brain, heart, lungs, colon, and kidneys, while TMPRSS2 expression has been found in tissues and organs such as the lungs, intestines, kidneys, and liver [13,14]. As a result, the expression of ACE2 and TMPRSS2 across organs may carry implications for the strategies aimed at preventing or treating SARS-CoV-2.

**Figure 1:**
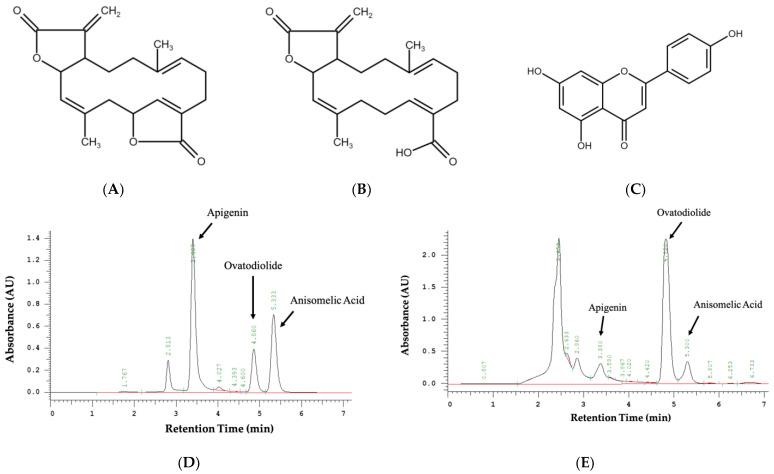
The chemical structures of *A*. *indica* active compounds: (**A**) ovatodiolide, (**B**) anisomelic acid, and (**C**) apigenin and chromatographic profile of *A. indica* using HPLC. HPLC chromatograms of (**D**) ovatodiolide standard compounds, anisomelic acid standard compounds, and apigenin standard compounds, as well as (**E**) ethanolic extracts of *A. indica* (EEAI).

*Anisomeles indica* (L.) Kuntze (yu-jen-tsau), belonging to the Lamiaceae family, is a frequently used medicinal plant in folk medicine, renowned for its various pharmacological activities including anti-HIV [15], antibacterial [16], antioxidant [17], and anticancer properties [18,19]. It has shown significant potential in the development of antiviral [20], anti-inflammatory [21], and antitumor drugs [22]. According to the findings of the investigation, it has been determined that *A. indica* contains various active compounds, including terpenoids such as ovatodiolide and anisomelic acid (Figure 1A,B), as well as flavonoids such as apigenin (Figure 1C) [20,22,23,24]. Among them, research has demonstrated that ovatodiolide suppresses the TGF-β/TβRs signaling pathway, leading to the inhibition of SARS-CoV-2 replication and amelioration in pulmonary fibrosis [25]. Recent research has also indicated that oral administration of anisomelic acid can effectively suppress SARS-CoV-2 virus replication and alleviate virus-induced cellular pathology [20]. Other studies indicate that apigenin demonstrates antiviral properties by restraining the synthesis of viral coat proteins and disrupting the interaction of viral RNA with transcription factors [26]. In summary, we have identified the potential of the antiviral effects of *A. indica* as possible prophylactic or therapeutic agents against SARS-CoV-2 infection. Therefore, this research focuses on analyzing the modulation of ACE2 and TMPRSS2 protein expression levels via ethanolic extracts of *A. indica* (EEAI) and its constituent compounds, encompassing investigations conducted in vivo and in vitro. The primary goal of this research is to investigate the potential association between *A. indica* and its constituents and the downregulation of ACE2 and TMPRSS2. The ultimate aspiration is to establish a robust research foundation that could serve as valuable reference for studies on combating COVID-19.

## 2. Results

### 2.1. Determination of EEAI Constituents Using HPLC

Ovatodiolide, anisomelic acid, and apigenin were used as markers to identify *A. indica*. The constituents of *A. indica* were assessed using HPLC-PAD (photodiode array detection). Figure 1D and Figure S1A indicates that ovatodiolide can be distinguished by its specific retention time (4.8 min), as well as anisomelic acid (5.2 min) and apigenin (3.4 min). Figure 1E and Figure S1B shows the relative contents of ovatodiolide, anisomelic acid, and apigenin in 2500 μg/mL ethanolic extracts of *A. indica*, quantified at 679.27, 37.94, and 15.98 μg/mL. These values were calculated based on Figure S2 and Tables S1–S4.

### 2.2. Evaluating the Impact of EEAI on the Proliferation of HepG2 and HEK 293T Cell Lines

**Figure 2:**
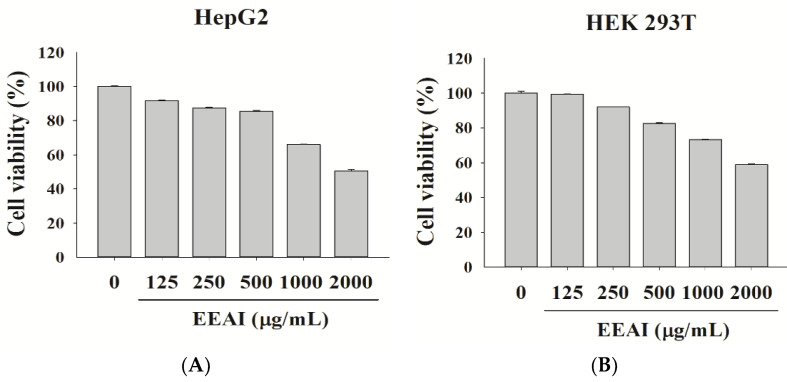
Cell viability of HepG2 cells and HEK 293T cells treated with EEAI. (**A**) HepG2 cells and (**B**) HEK 293T cells. The cells were treated with EEAI at different concentrations (125–2000 μg/mL) for 24 h and their viability was evaluated through the MTT assay. The outcomes demonstrated were a product of a minimum of three independent trials.

ACE2 serves as a receptor for SARS-CoV-2, aiding the virus in binding, crossing the membrane, and entering the cell. SARS-CoV-2 is subjected to TMPRSS2-mediated modifications to penetrate cells. To investigate the effects of EEAI on ACE2 and TMPRSS2, different drug levels were applied (125–1000 μg/mL) to HepG2 and HEK 293T cells during the experimentation. A 3-(4,5-dimethylthiazol-2-yl)-2,5-diphenyltetrazolium bromide (MTT) assay was utilized to determine the cytotoxicity of EEAI toward cells in preparation for subsequent research. As shown in Figure 2, the results indicated that EEAI at 250 and 500 μg/mL concentrations did not induce toxicity towards HepG2 and HEK 293T cells, so 250 and 500 μg/mL concentrations were chosen for subsequent experiments.

### 2.3. Investigating the Impact of EEAI on ACE2 and TMPRSS2 Expression Levels in HepG2 and HEK 293T Cell Lines

**Figure 3:**
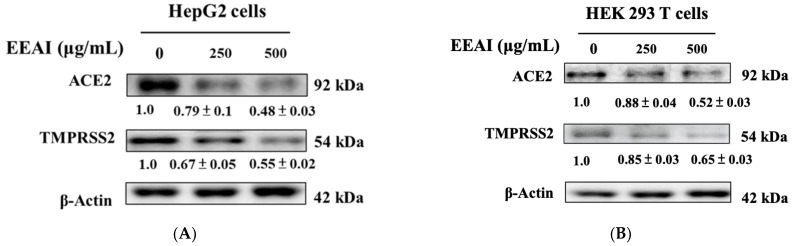
ACE2 and TMPRSS2 expression levels in HepG2 cells and HEK 293T cells upon EEAI treatment. Different concentrations of EEAI (250 and 500 μg/mL) were used to treat HepG2 (**A**) and HEK 293T (**B**) cells, which were cultured for a duration 24 h. The analyses of ACE2 and TMPRSS2 expression were conducted using Western blotting. Densitometric analysis was conducted and the results were depicted as a ratio (EEAI/control), using β-actin as an internal control.

After evaluating the impact of EEAI on cell lines, we confirmed the function of EEAI in modifying ACE2 and TMPRSS2 protein expression levels. Figure 3 suggests that after 24 h treatment using EEAI, there was a marked dose-responsive reduction in ACE2 and TMPRSS2 protein expression levels in HepG2 cells and HEK 293T cells. In HepG2 cells, the expression levels of ACE2 and TMPRSS2 decreased by 21–52% and 33–45%, respectively, depending on the concentration of EEAI. Similarly, in HEK293T cells, there were corresponding decreases of 12–48% for ACE2 and 15–35% for TMPRSS2.

### 2.4. Evaluating the Impact of A. indica Constituents on the Proliferation of HepG2 and HEK 293T Cell Lines

**Figure 4:**
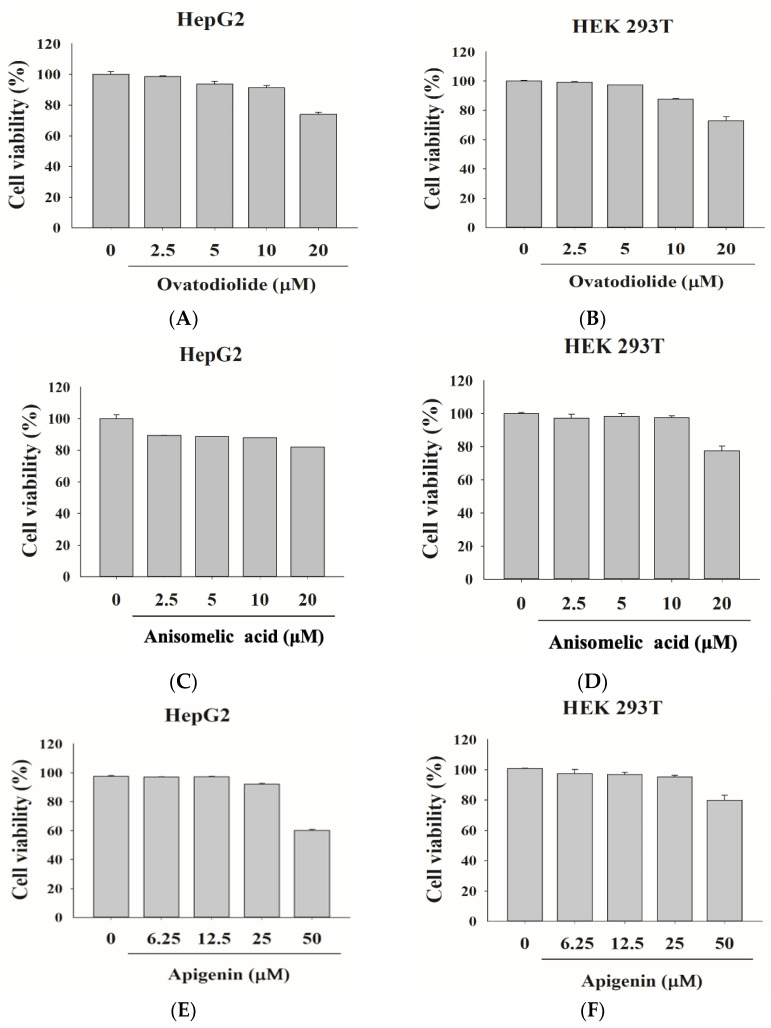
Cell viability of HepG2 cells and HEK 293T cells after ovatodiolide (**A**,**B**), anisomelic acid (**C**,**D**), and apigenin (**E**,**F**) treaments. The cells were treated with ovatodiolide (2.5–20 μM), anisomelic acid (2.5–20 μM), and apigenin (6.25–50 μM) at various doses for 24 h, and their viability was evaluated through an MTT assay. The outcomes shown are the product of a minimum of three independent trials.

Ovatodiolide, anisomelic acid, and apigenin are constituents extracted from EEAI. To examine the effects of these constituents on HepG2 and HEK 293T cells, we administered various concentrations of the drugs to the cells for investigation. Ovatodiolide (2.5–20 μM), anisomelic acid (2.5–20 μM), and apigenin (6.25–50 μM) were used for HepG2 cells. Ovatodiolide (2.5–20 μM), anisomelic acid (2.5–20 μM), and apigenin (6.25–50 μM) were used for HEK 293T cells. As the results in Figure 4 show, we measured the cell viability of the *A. indica* constituents in the cells using an MTT assay and determined the concentration of each constituent in subsequent experiments. Ovatodiolide (5 and 10 μM), shown in Figure 4A, anisomelic acid (5 and 10 μM), shown in Figure 4C, and apigenin (12.5 and 25 μM), shown in Figure 4E, were selected for use in HepG2 cells. Ovatodiolide (5 and 10 μM), shown in Figure 4B, anisomelic acid (5 and 10 μM), shown in Figure 4D, and apigenin (12.5 and 25 μM), shown in Figure 4F, were selected for HEK 293T cells.

### 2.5. Investigating the Impact of A. indica Constituents on ACE2 and TMPRSS2 Expression Levels in HepG2 and HEK 293T Cell Lines

**Figure 5:**
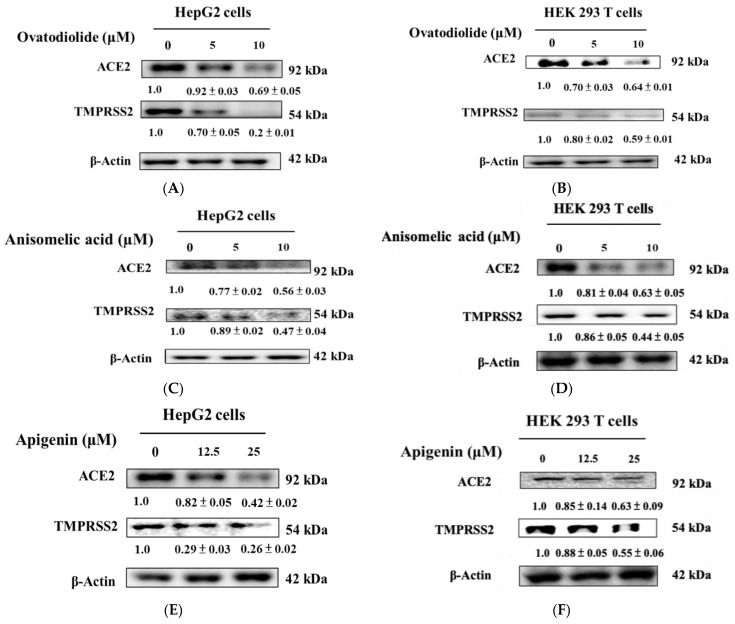
ACE2 and TMPRSS2 expression in HepG2 cells and HEK 293T cells upon ovatodiolide, anisomelic acid, and apigenin treatment. The HepG2 cells and HEK 293T cells were treated with different concentrations of ovatodiolide (**A**,**B**), anisomelic acid (**C**,**D**), and apigenin (**E**,**F**) and were cultured for a duration of 24 h. The analyses of ACE2 and TMPRSS2 expression were conducted using Western blotting. Densitometric analysis was conducted and the results are depicted as a ratio (EEAI/control), utilizing β-actin as an internal control.

After evaluating the impacts of ovatodiolide, anisomelic acid, and apigenin on cell lines, we validated their ability to alter protein expression levels of ACE2 and TMPRSS2. Figure 5 illustrates the results, which indicate that after 24 h of treatment with ovatodiolide (A), anisomelic acid (B), and apigenin (C), there was a notable decrease in ACE2 and TMPRSS2 protein expression levels in HepG2 cells and HEK 293T cells. In HepG2 cells, the expression of ACE2 decreased by 8–31%, 23–44%, and 18–58% with ovatodiolide, anisomelic acid, and apigenin, respectively, while the expression of TMPRSS2 decreased by 30–80%, 11–53%, and 71–74% for the corresponding compounds. In HEK293T cells, ACE2 expression decreased by 30–36%, 19–37%, and 15–37%, while TMPRSS2 expression decreased by 20–41%, 14–56%, and 12–45% for the corresponding compounds.

### 2.6. Evaluating the Impact of EEAI in Animal Testing

**Figure 6:**
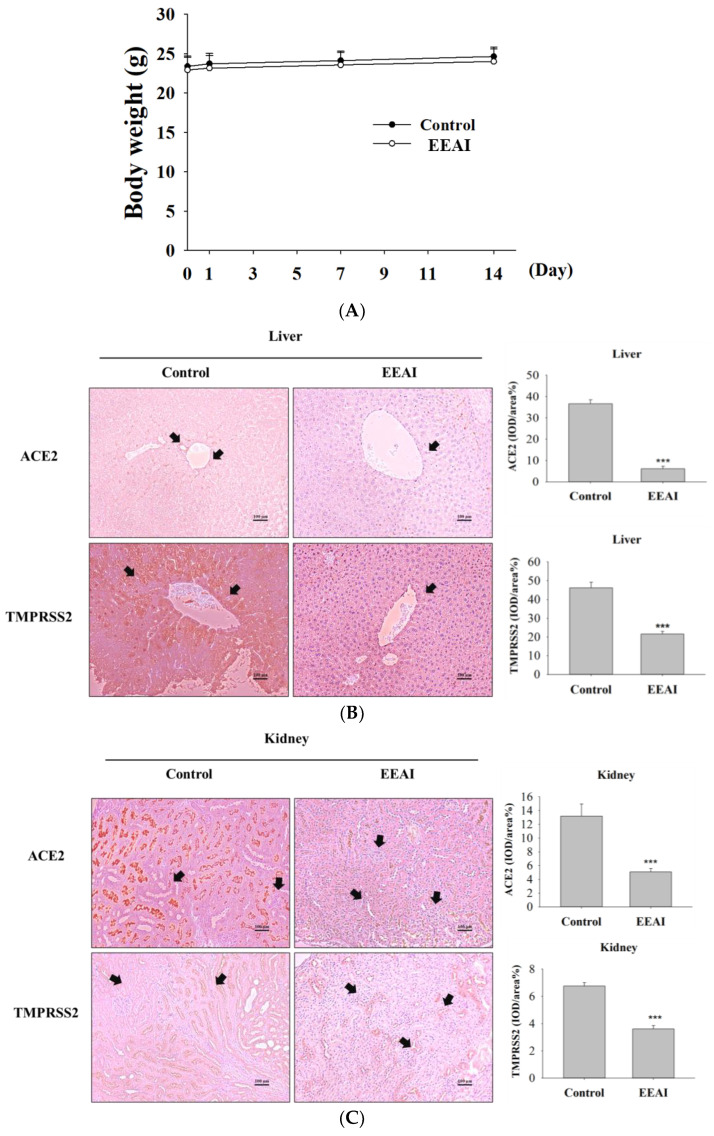

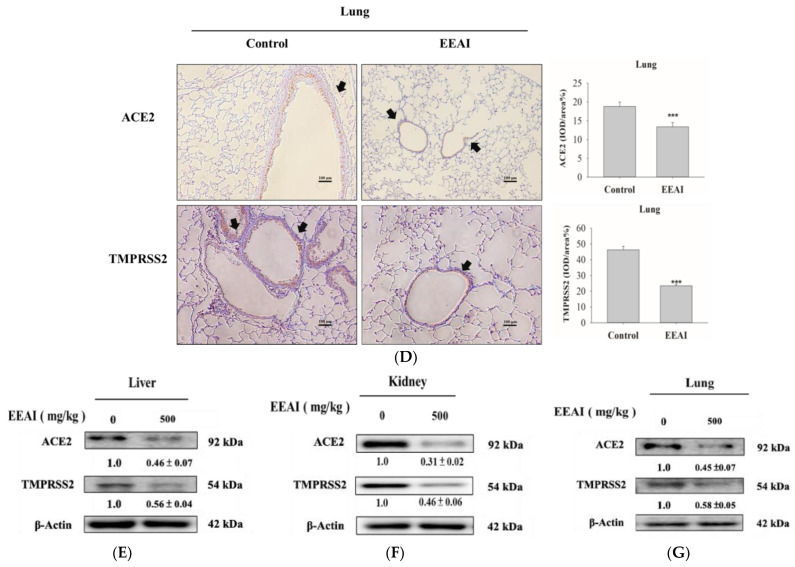
The impact of EEAI in animal testing. (**A**) The mice’s weights and counts were assessed subsequent to an oral gavage with 500 mg/kg EEAI. Images were derived from immunohistochemical staining of (**B**) liver, (**C**) kidney, and (**D**) lung tissue. Following IHC staining, histological sections were enlarged to 200× and photographed for the record. Results were showcased using IOD/area (%) measurements. Mean ± SD values (*n* = 6) are provided. *** *p* < 0.001 indicate significant differences compared to the control group. ACE2 or TMPRSS2 expression is marked by arrows (scale bar = 100 µm). The ACE2 and TMPRSS2 expression levels were assessed using Western blotting in (**E**) liver, (**F**) kidney and (**G**) lung tissues after treatment with 500 mg/kg EEAI, utilizing β-actin as an internal control.

To investigate the in vivo effects of EEAI, we conducted a mouse model experiment. As shown in Figure 6A, mice received a treatment of 500 mg/kg EEAI for a duration of 14 days. The mice’s body weights remained relatively stable over the course of 14 days.

### 2.7. Evaluation of In Vivo ACE2 and TMPRSS2 Expression through Immunohistochemical (IHC) Analysis

Figure 6B–D illustrates the results of the IHC analysis, which indicated abundant stained cells in the control group, while the EEAI (500 mg/kg) group demonstrated a marked reduction in ACE2 and TMPRSS2 expression in the tissues of the liver (Figure 6B), kidney, and lung (Figure 6C,D). These investigations revealed that EEAI can inhibit ACE2 and TMPRSS2 expression in the liver, kidney, and lung, while also maintaining a lack of liver, renal, or pulmonary toxicity.

### 2.8. Investigating ACE2 and TMPRSS2 Protein Expression Levels In Vivo

For the purpose of verifying the lowered ACE2 and TMPRSS2 expression attributed to EEAI, Western blotting was carried out. The protein expression levels of ACE2 and TMPRSS2 in the liver, kidney, and lung tissues of mice were markedly reduced as a result of *A. indica* treatment, as indicated by the results in Figure 6E–G.

## 3. Discussion

Towards the end of 2019, the COVID-19 pandemic began with the appearance of a novel coronavirus, SARS-CoV-2, in Wuhan. From there, it swiftly propagated across the globe, leading to widespread transmission and affecting numerous regions worldwide [27,28]. As of March 2023, it has resulted in over 764 million confirmed cases globally, with a cumulative death toll of 6.8 million cases [29]. COVID-19-infected patients have the potential to suffer from serious complications and organ damage, such as lung and kidney injuries, as well as systemic immune dysregulation [2,8,30,31,32]. Furthermore, after the rapid onset of the ailment, many patients have manifested post-acute sequelae of SARS-CoV-2 infection (PASC), with common symptoms including memory loss, fatigue, muscle and joint pain, and even psychological health disorders such as anxiety or depression. Consequently, considering the vast multitude of people affected by COVID-19 and the subsequent emergence of PASC, this ailment has left a substantial imprint on global public health [33]. Preventing and treating SARS-CoV-2 is both urgent and crucial.

Based on research observations, it is evident that there is a strong connection between SARS-CoV-2 infection and ACE2 and TMPRSS2. ACE2 primarily acts as a critical factor in maintaining the stability of the renin–angiotensin–aldosterone system (RAAS) in the human body. This system regulates functions such as vascular constriction and blood pressure, as well as cardiovascular and renal functions [9]. Moreover, ACE2 serves as a critical receptor that enables SARS-CoV-2 to invade human cells by attaching to its spike protein [10,27,34]. TMPRSS2, which functions as a serine protease, on the other hand, serves as a pivotal facilitator of the entry and activation of SARS-CoV-2 by cleaving its spike protein [11,35]. Moreover, through molecular docking studies, researchers have discovered that the interaction between SARS-CoV-2 and human ACE2 is strengthened in terms of affinity compared to SARS-CoV. The heightened binding efficiency of SARS-CoV-2 with human ACE2 also contributes to increased virus transmission among individuals, demonstrating the robustness of SARS-CoV-2’s spike protein in binding to human ACE2, which facilitates cellular infection through interactions with ACE2 receptors within the body. Therefore, reducing the expression levels of ACE2 and TMPRSS2 is crucial for preventing or treating SARS-CoV-2 infection. In animal experiments, researchers have also discovered that in mouse models infected with SARS-CoV, higher levels of ACE2 expression are associated with greater disease severity [36,37]. This observation highlights the crucial role of the virus’s entry into cells. Consequently, reducing ACE2 expression within the body can indirectly alleviate illnesses caused by the SARS-CoV-2 virus, potentially leading to effective treatments for COVID-19.

Among the results of this study, administration of the *A. indica* extract EEAI orally to mice led to a significant reduction in ACE2 and TMPRSS2 expression levels in liver, kidney, and lung tissues, as observed through immunohistochemical (IHC) analysis. Additionally, the measurements of ACE2 and TMPRSS2 protein expression levels showed consistent results. Following treatment with EEAI, a notable decrease in the ACE2 and TMPRSS2 expression levels was observed in the aforementioned mouse organ tissues, indicating the downregulation of ACE2 and TMPRSS2 by *A. indica* and suggesting that it have the potential to achieve therapeutic and preventive effects against SARS-CoV-2.

In vitro experiments in this study employed two cell lines, HepG2 and HEK293T. HepG2 was chosen for its rapid proliferation characteristics while retaining the genotypic and phenotypic features of normal cells [38]. Moreover, it possesses lower metabolic capacity and has been demonstrated to assess the toxicity of 93% of compounds [39]. Furthermore, compared to animal cell lines such as CHO-k1 and ECC-1, HepG2 cells can better predict human cell responses [39,40]. Hence, the HepG2 cell line has extensive applications in cell toxicity experiments. On the other hand, the HEK293T cell line has been utilized in research related to mitochondria and antiviral drugs, cell apoptosis, and glucose transport proteins [41,42]. Considering these factors, both of these cell lines were chosen for our experimental work.

Previous studies have indicated that in vitro experiments using cell cultures of *Sambucus nigra* effectively suppress the interaction of SARS-CoV-2 and ACE2 [43]. Additionally, other research has found that glycyrrhizin similarly demonstrates the suppression of the connection between SARS-CoV-2’s spike protein and ACE2 in in vitro experiments [44]. *Schizophyllum commune* has been shown to downregulate both ACE2 and TMPRSS2 expression, thereby inhibiting the entry of SARS-CoV-2 into cells [45]. Furthermore, in this study, following treatment with *A. indica* extract EEAI, both the HepG2 and HEK293T cell lines exhibited a significant decrease in the protein expression levels of ACE2 and TMPRSS2. Therefore, these in vitro experiments further validate the potential of *A. indica* to downregulate the expression levels of ACE2 and TMPRSS2.

*A. indica* is a commonly used medicinal plant in traditional medicine, known for its various pharmacological activities, including anti-HIV, antibacterial, antioxidant, and anticancer properties. It has shown potential in drug development for antiviral and anti-tumor agents [16,17,18,19,46,47,48]. *A. indica* is rich in various active compounds, such as ovatodiolide, anisomlic acid, and apigenin, all of which have been indicated by studies to possess antiviral effects. Among them, recent studies have also indicated that ovatodiolide and anisomlic acid have been found to inhibit the replication of the SARS-CoV-2 virus [20,25].

The in vitro experiments conducted in this study demonstrated that HepG2 and HEK293T cell lines exhibited a significant reduction in the protein expression levels of ACE2 and TMPRSS2 when treated with the active compounds of *A. indica*, which include ovatodiolide, anisomelic acid, and apigenin, at proportional dosages. This observation is consistent with the results obtained from the treatment with *A. indica* extract EEAI, suggesting that the presence of these three specific components in *A. indica* might contribute to the inhibition of ACE2 and TMPRSS2 expression.

In accordance with the results mentioned in this study, both in vivo experiments involving IHC and Western blotting analysis consistently demonstrated that *A. indica* extract EEAI effectively downregulated ACE2 and TMPRSS2 protein expression levels in the liver, kidneys, and lungs of mice. These findings were further corroborated by in vitro experiments. Additionally, research focusing on ovatodiolide, anisomelic acid, and apigenin also indicated that *A. indica*’s significant reduction in ACE2 and TMPRSS2 expression could be attributed to the presence of these three components. Therefore, considering the above research, we regard *A. indica* as a potential contender for easing the extent of COVID-19 infection’s seriousness.

## 4. Materials and Methods

### 4.1. Materials

The whole herb of *Anisomeles indica* (L.) Kuntze was provided by ARJIL Pharmaceuticals LLC Ltd. in Hsinchu, Taiwan. First, the 1000 g whole of the *A. indica* herb was soaked in 5000 mL 95% ethanol (ECHO chemical CO., LTD., Taichung, Taiwan) and extracted in a 60 °C bath for 4 h, then concentrated under reduced pressure to obtain the *A. indica* extract of EtOH (EEAI). This study used HPLC to determine the components and active components of EEAI extracts, which were used for subsequent experiments.

### 4.2. Quantification of EEAI Components via HPLC

HPLC analysis was employed to determine the composition of EEAI. The eluted fractions were characterized based on their retention time in comparison to the reference standard ovatodiolide (ARJIL Pharmaceuticals LLC Ltd., Hsinchu, Taiwan), anisomelic acid (ARJIL Pharmaceuticals LLC Ltd., Hsinchu, Taiwan), and apigenin (Chengdu Must Bio-Technology Co., Ltd., Chendu, China). The constituents were characterized by utilizing a photodiode array detector and comparing them with standard UV spectra at a wavelength of 220 nm. A TSK gel Tosoh ODS-80Tm column (250 × 4.6 mm i.d., 5 µm) (Tosoh, Yamaguchi, Japan) in reversed phase was employed for compound separation. During the interval of 0–8 min, a mixture of acetonitrile (J.T Baker, Phillipsburg, NJ, USA) and 0.1% acetic acid (Cascina Favaglie, Milan, Italy) in water (64:36) was used as the mobile phase while keeping the flow rate consistent at 1 mL/min [49].

### 4.3. Cultivation and Treatment of Cells

The HepG2 cell line (human hepatocellular carcinoma) and the HEK 293T cell line (human embryonic kidney) were procured from the Bioresource Collection and Research Center (Taiwan) and were put to use in this study. Cell cultures were regularly maintained in Dulbecco’s Modified Eagle Medium (DMEM) containing 10% fetal bovine serum (FBS) incubated at 37 °C with 5% CO_2_. For the following experiments, the concentration of compounds was carried out using the culture medium. In each well of a six-well tissue culture plate, 2.5 × 10^5^ cells per mL were placed for seeding. Following the predetermined 24 h of treatment, the collected samples were lysed using RIPA buffer. After that, the supernatant obtained was processed for purification through centrifugation at 10,000× *g* and 4 °C for a period of 15 min in a refrigerated centrifuge. Stored at −20 °C, it was employed in further experimentation.

### 4.4. 3-(4,5-Dimethylthiazol-2-yl)-2,5-diphenyltetrazolium Bromide (MTT) Assay

HepG2 and HEK293T cells were seeded into 96-well tissue culture plates, with each well receiving 2.5 × 10^5^ cells per mL, incorporating 10% FBS into DMEM. The plates were then placed in an incubator set at 37 °C and 5% CO_2_ for a duration of 24 h.

Once cell attachment was observed, the medium was renewed with a 10% FBS-enriched fresh medium. Subsequently, the medium was further supplemented with the appropriate drug concentrations and incubated for a 24 h duration. After this step, we removed the supernatant. Following this, 100 μL of DMEM containing 0.5 mg/mL MTT reagent was added to the cell culture and incubation was carried out for at least 3 h. Following this, this supernatant was discarded and 100 μL of DMSO was introduced. Subsequent to the 10 min interval, cell viability was gauged through absorbance measurements at 570 nm using an ELISA reader.

### 4.5. Western Blotting

The Bio-Rad protein assay kit was employed to measure the total protein concentration. In the electrophoresis process, 20 µg of proteins was loaded into each well and subsequently separated on a gel before being transferred onto a PVDF membrane. A mixture comprising 3–5% non-fat milk and TBST was prepared, to be used for blocking the blank area for at least 1 h before binding the primary antibody (ACE2 1:1500:GTX101395; TMPRSS2 1:1500:GTX100743, Genetex, San Antonio, TX, USA) to the target protein and incubating at 4 °C overnight. On the following day, after the primary antibody eliminated and rinsed it with TBST, the secondary antibody (goat anti-rabbit IgG antibody (HRP) 1:5000: ARG65351; Arigo, Hsinchu, Taiwan) was added to enhance the recognition signal to detect the target protein. After a series of treatments, to magnify the signal, horseradish peroxidase (HRP) conjugate and ECL substrate (201765; Merck, Branchburg, NJ, USA) were utilized. Ultimately, the signals were captured through the use of Kodak Gel Logic 1500 Imaging Software version 4.0 (East-man Kodak Company, Rochester, NY, USA).

### 4.6. Animal Model

BioLASCO (Taipei, Taiwan) provided us with 12 male C57BL/6 mice aged 6–8 weeks and weighing 18–20 g for the study. The mice were randomly distributed into two groups (*n* = 6) each. Then, 500 mg/kg EEAI dissolved with distilled water was administered to the treatment group through oral gavage over a ten-day period, in contrast to the control group, which received routine treatment. On Day 0, Day 1, Day 7, and Day 14, the weights of the mice were documented. Following a fourteen-day period, the mice were sacrificed, and samples of whole blood, liver, kidney, and lung were obtained.

### 4.7. Histopathological Examination

After embedding in paraffin, visceral tissues were sectioned into 3 µm layers and then treated with hematoxylin–eosin staining (H&E) to facilitate visualization. Microscopic examination was conducted on liver, kidney, and lung tissue sections (Nikon, ECLIPSE, TS100, Tokyo, Japan), followed by capturing images using a microscope camera (Jenoptik, ProgRes CF Scan, Fremont, CA, USA).

### 4.8. Immunohistochemistry (IHC) Analysis

The embedded visceral tissues were sectioned into 3 µm segments, followed by immunohistochemistry to stain the target. ACE2 primary antibody (bs-1004R, Bioss Inc., Woburn, MA, USA, dilution 50×) or TMPRSS2 primary antibody (ab214462, Abcam, dilution 200×) was used to stain liver, kidney, and lung tissue samples from the mice. The IHC assessment was performed utilizing a Polink-2 Plus HRP DAB Rabbit Bulk kit (D39, GBI LABS) in accordance with the guidelines provided by the manufacturer. The observations were conducted using a Nikon microscope (ECLIPSE, TS100, Japan), and images were documented by microscope camera (Jenoptik, ProgRes CF Scan, Fremont, CA, USA).

### 4.9. Statistical Analysis

Mean values ± standard deviation (SD) were depicted for all data using SPSS software version 21.0 (SPSS, Inc., Chicago, IL, USA). For comparing two groups, an unpaired two-tailed Student’s *t*-test was employed along with an unpaired two-tailed Student’s *t*-test, whereas one-way analysis of variance (ANOVA) followed by Scheffé’s test was employed for analyses involving more than two groups. The threshold for statistical significance was set at *p*-values less than 0.05.

## 5. Conclusions

Throughout the course of this investigation, we conducted both in vivo and in vitro experiments, which revealed that ethanolic extracts of *A. indica* and its components (ovatodiolide, anisomelic acid, and apigenin) effectively reduce the expression levels of ACE2 and TMPRSS2. These findings were consistent across HepG2 and HEK 293T cell lines as well as in a mouse model, as confirmed by Western blotting. The IHC analysis of mouse liver, kidney, and lung tissues also yielded similar results. It is crucial to note that the entry of SARS-CoV-2 into cells is closely associated with ACE2 and TMPRSS2. By modulating the expression levels of ACE2 and TMPRSS2, we can effectively inhibit SARS-CoV-2 infection, thereby contributing to the prevention and treatment of COVID-19. Therefore, we firmly believe that ethanolic extracts of *A. indica* and its constituents hold great promise as potential drugs for combating SARS-CoV-2 infection. This discovery provides a solid foundation for further research and development, offering new preventive and therapeutic options for the global fight against the COVID-19 pandemic.

## Data Availability

Data are contained within the article.

## Supplementary Materials

The following supporting information can be downloaded at: https://www.mdpi.com/article/10.3390/ijms242015062/s1.
