# Supplementary material for: Anisomeles indica Extracts and Their Constituents Suppress the Protein Expression of ACE2 and TMPRSS2 In Vivo and In Vitro
Source: Int J Mol Sci. 2023 Oct 11;24(20):15062. doi: 10.3390/ijms242015062 (PMC10606724; doi:10.3390/ijms242015062)
Supplement: Supplementary file 1 [file ijms-24-15062-s001.zip › ijms-2615744-supplementary.pdf]

The qualitative and quantitative analysis of *Anisomeles indica* by HPLC

The following detailed account provides a qualitative and quantitative analysis of *Anisomeles indica*.

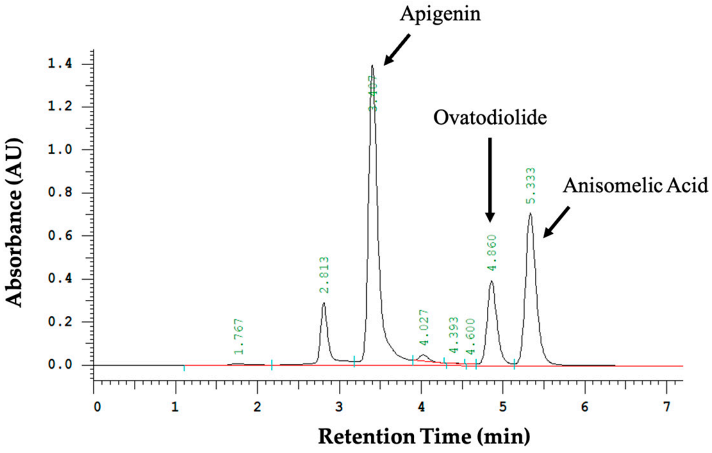

(**A**)

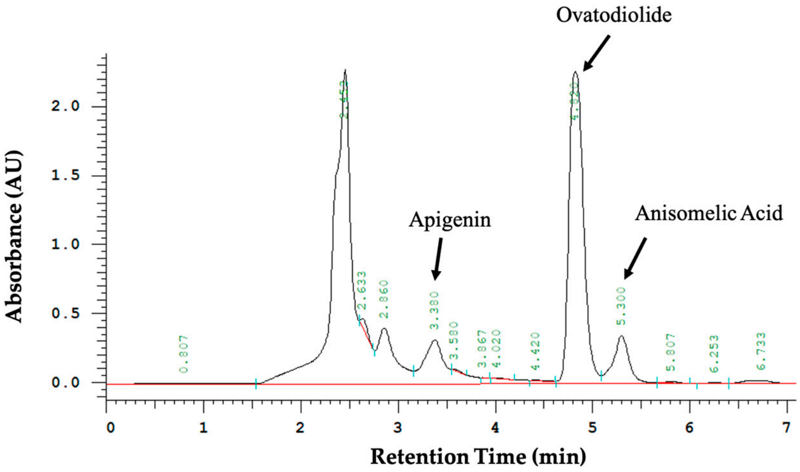

(**B**)

Figure S1. Chromatographic profile of *A. indica* using HPLC. HPLC chromatograms of (**A**) Ovatodiolide standard compounds, Anisomelic acid standard compounds and Apigenin standard compounds and (**B**) ethanolic extracts of *A. indica* .

In the qualitative analysis, three reference standards, ovatodiolide, anisomelic acid, and apigenin, were individually subjected to HPLC analysis at a concentration of 100 μg/mL, as shown in Figure S1A. Ovatodiolide can be distinguished by its specific retention time (4.8 min), anisomelic acid (5.2 min), and apigenin (3.4 min). In Figure S1B, *A. indica* exhibits the presence of identical

components at corresponding retention times In the quantitative analysis, gradient dilutions of ovtodiolide, anisomelic acid, and apigenin were prepared at concentrations of 1000 μg/mL, 500 μg/mL, and 125 μg/mL, respectively, to establish calibration curves, as depicted in Figure S2. Detailed peak data of HPLC can be found in Tables S1 and S3.

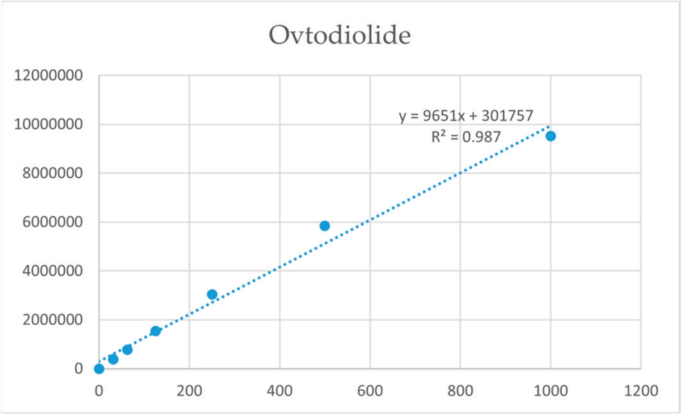

(**A**)

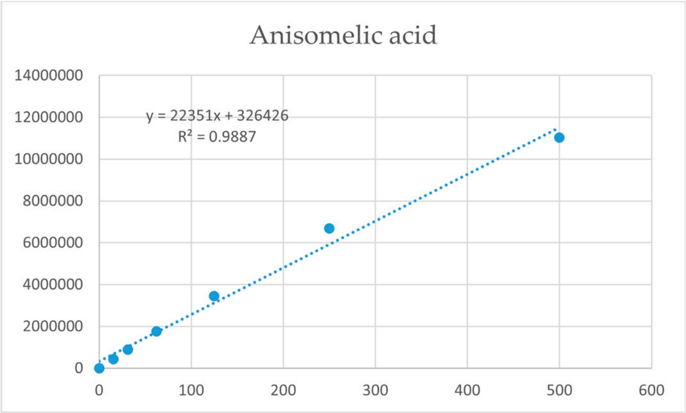

(**B**)

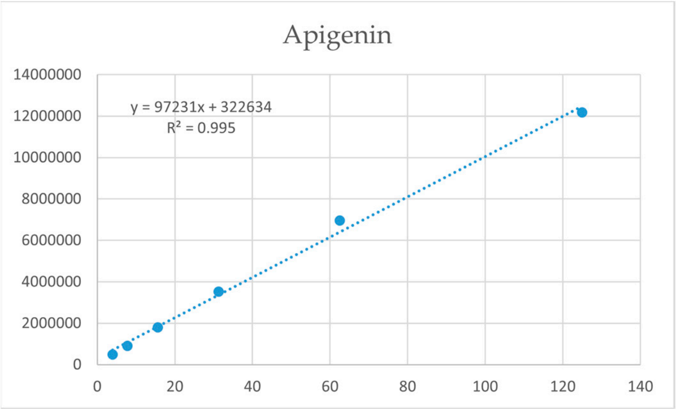

(**C**)

Figure S2. The calibration curves for the three reference standards. (**A**) Ovatodiolide standard compound, (**B**) anisomelic acid standard compound and (**C**) apigenin standard compound.

Tables S1. The peak area of Ovatodiolide calibration curves

Ovatodiolide	retention time	concentration (μg/mL)	peak area (triplicate)
STD6	4.827	1000	10192820
			8269283
			10112913
STD5	4.827	500	5873449
			5764379
			5884137
STD4	4.827	250	3095871
			2993061
			3025263
STD3	4.827	125	1573825
			1542694
			1529082
STD2	4.827	62.5	784245
			761566
			777405
STD1	4.827	31.25	385678
			383879
			388272
blank	0	0	0

Tables S2. The peak area of Anisomelic acid calibration curves

Anisomelic acid	retention time	concentration (μg/mL)	peak area (triplicate)
STD6	5.307	500	11808069
			9565276
			11746932
STD5	5.307	250	6730817
			6617164
			6729299
STD4	5.313	125	3528225
			3398760
			3434567
STD3	5.307	62.5	1785788
			1748555
			1742205
			915710
			877193

STD2	5.307	31.25	910365
STD1	5.307	15.625	429191
			429697
			462552
blank	0	0	0

Tables S3. The peak area of Apigenin calibration curves

Apigenin	retention time	concentration (μg/mL)	peak area (triplicate)
STD6	3.387	125	13259595
			10163993
			13104636
STD5	3.387	62.5	6963609
			6882838
			7032232
STD4	3.387	31.25	3587647
			3481622
			3529173
STD3	3.387	15.625	1832356
			1812776
			1772583
STD2	3.387	7.8125	913219
			888654
			902422
STD1	3.387	3.90625	451263
			472044
			540516
blank	0	0	0

This study employed quantitative analysis using 2500 μg/mL ethanolic extracts of *A. indica*. The corresponding peak areas in the HPLC are shown in Table S4. After averaging the peak areas of the three components, they were individually substituted into the respective calibration curve formulas to obtain the relative concentrations.

The calibration curve formulas of three reference standards:

Ovatodiolide: y = 9651 x + 301757
Anisomelic acid: y = 22351 x + 326426
Apigenin: y = 97231 x + 322634

The relative content of ovatodiolide, anisomelic acid, and apigenin is 679.27, 37.94, and 15.98 μg/mL, respectively.

Tables S4. The peak area of 2500 μg/mL ethanolic extracts of *A. indica* is presented as triplicate measurements

	Ovatodiolide	Anisomelic acid	Apigenin
2500μg/mL-1	6807886	1165976	1926968
2500μg/mL-2	6787731	1117184	1769947
2500μg/mL-3	6976518	1239838	1931537
Average	6857378	1174333	1876150.7
